# The Hospital Microbiome Project: Meeting Report for the 1st Hospital Microbiome Project Workshop on sampling design and building science measurements, Chicago, USA, June 7th-8th 2012

**DOI:** 10.4056/sigs.3717348

**Published:** 2013-04-15

**Authors:** Daniel Smith, John Alverdy, Gary An, Maureen Coleman, Sylvia Garcia-Houchins, Jessica Green, Kevin Keegan, Scott T. Kelley, Benjamin C. Kirkup, Larry Kociolek, Hal Levin, Emily Landon, Paula Olsiewski, Rob Knight, Jeffrey Siegel, Stephen Weber, Jack Gilbert

**Affiliations:** 1 Argonne National Laboratory, 9700 South Cass Avenue, Argonne, IL 60439, USA; 2 Department of Surgery, University of Chicago, Chicago, IL, 60637, USA; 3 Department of Geophysical Sciences, University of Chicago, Chicago, IL, 60637, USA; 4 University of Chicago UCMC Infection Control, Chicago, IL, 60637, USA.; 5 Institute of Ecology and Evolution, University of Oregon, Eugene, OR, 97403, USA; 6 Department of Biology, San Diego State University, San Diego, CA 92182, USA; 7 Department of Wound Infections, Walter Reed Army Institute of Research, Silver Spring MD, 20910, USA; 8 FE Hébert School of Medicine, Uniformed Services University of the Health Sciences, Bethesda MD, 20814 USA; 9 Department of Pediatrics, Division of Infectious Diseases, Ann and Robert H. Lurie Children’s Hospital of Chicago, Chicago, IL 60611, USA; 10 The Building Ecology Research Group, Santa Cruz, CA, 95060, USA; 11 University of Chicago Medical Center, Chicago, IL, 60637, USA.; 12 Alfred P Sloan Foundation, New York, NY, 10111, USA.; 13 Howard Hughes Medical Institute and Department of Chemistry & Biochemistry, University of Colorado at Boulder, Boulder, Colorado, USA.; 14 Civil, Archectural and Environmental Engineering Department, University of Texas at Austin, Austin, TX, 78712, USA.; 15 Department of Ecology and Evolution, University of Chicago, Chicago, IL 60637, USA

## Abstract

This report details the outcome of the 1st Hospital Microbiome Project workshop held on June 7th-8th, 2012 at the University of Chicago, USA. The workshop was arranged to determine the most appropriate sampling strategy and approach to building science measurement to characterize the development of a microbial community within a new hospital pavilion being built at the University of Chicago Medical Center. The workshop made several recommendations and led to the development of a full proposal to the Alfred P. Sloan Foundation as well as to the creation of the Hospital Microbiome Consortium.

## Introduction

Hospital-acquired infections (HAI; also called nosocomial infections) are usually acquired between 48 hours and 4 days after a patient has been admitted to the hospital. Nosocomial infections currently affect 5% of patients admitted to medical facilities in the US, with the total number exceeding 1 million people – 1.7 million hospital acquired infections requiring more than 170 million patient days [1]. The implications of a diagnosed HAI are that a particularly transmissible or virulent organism either has a reservoir in the facility or is spreading from patient to patient in a susceptible population. These infections may be viral, bacterial, protozoan or fungal. The vast majority of these cases occur while the patient is being treated for the ailment that initially resulted in the hospital admission. Approximately 36% of these infections could be linked to lapses in infection control protocols, such as failure of healthcare workers to wash their hands after patient contact or through improper attention to the specific protocols for cleanliness in the hospital environment [8]. While these numbers are concerning, they also highlight a considerable lack of evidence regarding both the source and development of nosocomial infections. The workshop, held at University of Chicago on June 7th-8th, 2012, convened a working group comprising building design, construction, out-fitting professionals, as well as project managers, medical staff, hospital management, microbiologists, virologists, building scientists, and standards officials to define the most appropriate sampling regime to capture the source and development of microbial communities and specifically hospital-acquired infections in a new hospital building being built at the University of Chicago. This hospital became operational in February 2013, and the aim was to sample the building, patients, and staff in a systematic coordinated approach from January 2013 to January 2014.

Despite considerable interest in developing hospitals with reduced nosocomial infection rates, there has been no systematic analysis of the gradual development of pathogenic reservoirs over the course of the months following the opening of new healthcare facilities. Indeed, there is currently very limited direct evidence as to the source of the infections; if these are airborne then any transmission agent that interacts with the hospital environment could be a potential source of transmission. This is why it is essential to explore the systematic action of populating a hospital to at least qualitatively assess the mode of transmission. The primary hypotheses driving the design of the study are:

A. “The presence of a ‘beneficial’ or ‘human-benign’ microbiome on surfaces in a hospital ecosystem affects the rate of accumulation and the persistence of hospital acquired infective agents.” The *null*-hypothesis is that “the presence of existing diverse microbial communities will have no impact on increased nosocomial pathogen accumulation and persistence in the hospital.” *Testing this hypothesis will help to elucidate the interactions between members of microbial communities that influence the development of pathogen populations and potentially act to actively inhibit pathogen populations from colonizing hospital systems.*

B. “Nosocomial infections are linked to the occupancy patterns of patients that carry and transmit these infections, and the presence of the potential pathogens increases after patients are admitted to the hospital.” The *null*-hypothesis is that “patients are not the primary source for potential infective agents, instead the transient demographics and occupancy frequency of those demographic groups show complex interrelationships and sporadic disease development events”. *Testing this hypothesis will be fundamental to the experimental design, and will focus the human cohort sampling (i.e. who is swabbed, and when).*

C. “Species associated with nosocomial infections are more prevalent in indoor locations involving patient care”. The *null*-hypothesis is that “locations in the building not associated with patient care demonstrate the highest prevalence of nosocomial infective agents.” *Determining the most appropriate locations in which to sample so that this hypothesis can be adequately tested was the focus of the discussions with architects, project managers (including building and component engineers), building crews, custodial and medical staff (including the chief of epidemiology in the hospital – Emily Landon) on where and when samples should be acquired in the building*

The primary driver for the working group was to generate a plan for a spatial and temporal survey of the hospital building and personnel frequenting the building both before and after the start of medical activity. Determining the appropriate strategy to systematically characterize the potential changes in the building microbiota due to the natural evolution of microbial community succession associated with different built environments inside the building will enable the generation of datasets that could have considerable impact upon how hospitals are designed, constructed, and maintained after occupancy. Fundamentally, understanding these processes may impact hospital design, as well as population handling inside the hospitals, even those that are already built. A key aspect will be determining the influence of each human group on the microbial ecology of the building and the potential development of nosocomial reservoirs. Hence, exploring correlations of community profiles with information regarding the abundance and types of people using specific locations will help to determine the relationships between the development of the indoor microbiome in the hospital and the human social interaction with that space.

## Day 1

The first day of the workshop comprised the arrival, primary presentation and discussion forum for the meeting. The majority of people attended in person, except John Alverdy (Department of Surgery, University of Chicago, USA), Jessica Green (Department of Biology, University of Oregon, USA), Rob Knight (Departments of Chemistry & Biochemistry and Computer Science, University of Colorado at Boulder, USA), and Scott Kelley (Department of Biology, San Diego State University, USA). These four people attended remotely or periodically via the in-house television communication system. Those attending in person were: Jack Gilbert (Argonne National Laboratory, USA; Department of Ecology and Evolution, University of Chicago, USA), Daniel Smith (Institute for Genomic and Systems Biology, ANL, USA), CPT Benjamin C. Kirkup (Department of Wound Infections, Walter Reed Army Institute of Research, USA), Hal Levin (Building Ecology Research Group, USA), Jeffrey Siegel (Department of Civil, Architectural, and Environmental Engineering, University of Texas, USA), Kevin Keegan (IGSB, ANL, USA), Maureen Coleman (Physical Sciences Division, UC, USA), Sylvia Garcia-Houchins (Infection Control Program, University of Chicago Medical Center, USA), Larry Kociolek (Department of Pediatrics, Ann and Robert H. Lurie Children’s Hospital of Chicago, USA), Emily Landon (Department of Medicine, Infection Control, UCMC, USA), Stephen Weber (Clinical Excellence, UCMC, USA), Gary An (Department of Surgery, UCMC, USA), Julie Segre (Genetics and Molecular Biology Branch, National Human Genome Research Institute, USA), and Paula Olsiewski (Alfred P. Sloan Foundation, USA).

The meeting started with an introduction and welcome by Jack Gilbert, who provided an overview of the reason for the workshop. The rationale for the workshop was discussed in light of the initial workshop funding provided by the Alfred P. Sloan Foundation (APSF) to explore the potential to capture the microbial community succession in the new hospital pavilion being built in Chicago. It was agreed that, to explore this environment in collaboration with building scientists, microbiologists, ecologists and clinicians both prior to and following the introduction of patients and staff, was an opportunity that could not be wasted. Paula Olsiewski then gave an introduction and welcome on behalf of the APSF, providing a balanced introduction to why APSF was interested in funding projects to explore the indoor environment, and why explicitly they were interested in the opportunity of helping to fund a coordinated analysis of a hospital environment. It was noted that while the impetus of this endeavor had a definite medical application, APSF was not expressly interested in funding medical microbiology research.

Daniel Smith then started the meeting with a presentation on the initial vision for a hospital microbiome analysis. This presentation, which can be found on the hospitalmicrobiome.com website, describes the background for doing hospital microbiology research, the layout of the new hospital pavilion, and the need for studying the development of microbial communities in healthcare environments. It also outlined the ideal sampling strategy and a more realistic sampling strategy based on a modest financial funding opportunity. In the ideal model, 6,677 locations from hospital surfaces, air, water, and its occupants would be collected daily over the course of one year for a total of approximately 2.4 million samples. These would include 26 samples from the patient, air, and high-touch surfaces in each of the 240 patient rooms, as well as from hospital staff and surfaces outside the patient rooms, which see the most interactions with human traffic. However, because this level of comprehensiveness would cost $24 million in sample processing and sequencing alone, a smaller scale, more focused plan of action was proposed. Rather than sampling the entire hospital, the alternative plan called for selecting a representative group of 20 patient rooms, and tracking the airborne, waterborne, hostborne, and vectorborne microbial communities that come into contact with these rooms by collecting samples from 187 locations from patients, staff, water, air, and high-touch surface areas weekly for one year. Because collecting microbial samples is relatively inexpensive, and samples can be cryogenically preserved for long periods of time, a cost-saving strategy of this plan was to sample 20 rooms, but select only 8 for downstream processing and analysis based on rooms appearing to have high vs low rates of human-microbial interactions, as measured by diagnoses of hospital acquired infections. This sampling regimen, in conjunction with building science measurements (described below) and standard research expenses, was estimated to require approximately $300,000 in funding.

The timeline for this project was driven by the projected opening date for the new hospital pavilion – January 31st, 2013. Clinical cleanings designed to improve the level of cleanliness from construction site to hospital-grade will be completed by October 19th 2012. To attain a baseline measurement of variation in microbial community structure among the different elements in this project, the proposed timeline calls for beginning sampling of the new hospital pavilion and its future staff on January 1st, 2013. At that time, the future staff will be sampled in the current working location in order to observe changes in their microflora resulting from beginning work at the new hospital pavilion, and to acquire a “microbial fingerprint” of each staff member to be used in determining source contaminants of the new hospital pavilion. Observing the hospital surfaces, water, and air prior to the admission of patients is necessary for determining the rate at which microbial communities of the building change in the absence of human occupants. This rate can then be compared to rates of change for the occupied building on surfaces where patient microflora is postulated to be a driving factor of community change.

Several algorithms were put forward as useful tools for identifying patterns in community succession. SourceTracker [2] detects the spread of bacterial communities from one sample into another, for example soil tracked into patient rooms or water-borne bacteria onto hands. Microbial Assemblage Prediction (MAP) [3] identifies environmental variables, e.g. temperature, humidity, etc., that drive the rise or decline of microbial groups. Local Similarity Analysis [4] finds temporal patterns among community members, for example if particular taxa increases in abundance following a parallel increase in abundance of a different phylogenetic group.

The presentation prompted considerable discussion on two different fronts, led by two of the specific consortium partners, namely the building scientists and the clinicians. Here we breakdown some of the key findings from these discussions:

### Building science considerations

Hal Levin and Jeffrey Siegel represented the building science community at the workshop and provided extremely valuable input into the considerations regarding architectural design, building infrastructure and the need for appropriate measurements to define the specific aspects of the built environment that could have an impact on the microbial community diversity and structure. Sylvia Garcia-Houchins from the infection and control and immunity group at the University of Chicago Hospital was also extremely interested in building issues and highlighted the need to understand the key elements of pathogen reservoir development in the building. A lengthy discussion ensued regarding the water supplies and cooling towers as potential reservoirs of *Legionella spp*. infections. This led to agreement regarding the need to explore the water supply for the development of microbial communities and specific pathogens. In addition, the plumbing for the water system in the hospital was identified as a key concern, especially regarding the potential for ‘dead-ends’ in the water system on patient ward levels, which could act as potential pathogen breeding grounds. The issue of recording surface materials and cleaning schedules was also raised, and the need to determine the paint-types used on each surface. The need to record and measure the airflow and filtration system for both pressure, CO_2_ and microbial/viral community structure was also highlighted; the HVAC system in the hospital was discussed at length and it was agreed that the building schematics would be made available to explore the system in more detail. It was highlighted that ventilation systems, when new, are generally quite clean, but can rapidly become full of lint, dust and microorganisms that can cause significant hospital environmental contamination. The total building design was also taken into consideration including the use of ‘shell-floors’ on the lower part of the building, which were designed to remain empty for storage and potential expansion. These floors are enclosed but could act as potential reservoirs of microbial contaminants to the rest of the hospital. It was, however, decided that the risk of such infection was relatively minor, and these empty floors would not be considered for further analysis.

### Medical microbiology, clinical and epidemiological considerations

A particular point of concern in the proposed sampling methodology was the weekly sampling frequency. Though some patients reside in the same room for months, many other are admitted for only 1-3 days, potentially leaving behind their microflora without a biological record of its source. Further discussion suggested that hourly sampling may be necessary to comprehensively track fluctuations in microbial populations, though such fine resolution may be unnecessary for accomplishing the stated research objectives. Therefore to better balance comprehensiveness against replication within the target budget, the proposed methodology was amended to sample 2 rooms on a daily basis and the remaining 18 rooms on a weekly basis.

The selection of organisms to be monitored was another point of interest. The amplicon library strategy suggested in the presentation describes the presence of prokaryotic, eukaryotic, and fungal microorganisms by a 151 base pair segment of their genes encoding the small rRNA subunit. As a result, species-level identifications would be inconsistent [5], and the presence of antibiotic resistance genes would not be tracked. To more thoroughly describe the microbial populations relevant to healthcare settings, the use of metagenomics, antibiotic-resistance gene microarrays [6], culturing, and inclusion of viral assays were suggested. As a means to limit concomitant increases in expenses, such additional methods could be employed only on specific samples of interest.

Emily Landon brought to the group’s attention the possibility of tapping into the new hospital pavilion’s RFID system to track the movement of staff and visitors among the patient rooms. These data would allow microbial source tracking to be independently substantiated. For instance, data derived from rRNA amplicon libraries indicating that bacteria in room B originate from room A may be confirmed with RFID data showing that hospital staff regularly walk directly from room A to room B. The RFID system could also double as a monitor of room occupancy, which is a crucial metric for the building science calculations (described below) for computing the rate at which microorganisms are introduced into the environment through human respiration, as well as CO_2_ concentrations used to estimate the percentage of recycled air.

The importance of recording the behaviors and habits of hospital staff was also raised. Hand-washing is perhaps the single most effective measure in preventing hospital-acquired infections. Glove use was mentioned as having limited utility in preventing cross-contamination due to the fact that when reaching into a glove box for a pair of disposable glove, the skin contacts the outer layers of many hospital exam gloves, rendering them contaminated. Glove boxes were therefore posited as a relevant sampling location for this project. For these reasons, patient care can strongly impact the likelihood of transmission of infectious agents. Tracking, or asking staff to self-report, their degree of caution in limiting the spread of bacteria may be useful for quantifying hospital staff as vectors of bacterial communities.

Following the afternoon discussions, the group broke for dinner.

## Day 2

The second day of the workshop was predominated by a visit to the hospital building site to view the locations we had discussed in the afternoon of Day 1. Elizabeth Lockwood, the program manager, provided the group with a detailed tour of the building, starting with the cooling towers and air intake systems on the roof, and then working down through the building examining each of the floors. To provide an interesting ‘first-look’ at the hospital microbiome, Jack Gilbert and Daniel Smith worked with the group to sample the shoes of randomly chosen members of the team both before and after the tour, the floors, water supply, walls, taps, handles, control panels and equipment in the patient rooms, nurse stations, and corridors of the hospital during the tour. These samples have been DNA extracted, 16S rRNA amplified and sequenced, and the results published on the hospital microbiome website [7]. The tour was very successful at providing the attendees with real world experience of where the sampling would take place, and having clinicians, microbial ecologists and building scientists present provided ample opportunity for extensive cross-talk that helped to answer questions and augment the proposed research plan during and after the meeting.

### Wrap-up

It was agreed that another meeting be held in early 2013 during the first phase of the expected APSF funded research project to explore the evolution of the consortium, and the research agenda.
